# Step further towards targeted senolytic therapy: therapeutic potential of uPAR-CAR T cells for senescence-related diseases

**DOI:** 10.1038/s41392-020-00268-7

**Published:** 2020-08-13

**Authors:** Yujia Huang, Tao Liu

**Affiliations:** 1 State Key Laboratory of Natural and Biomimetic Drugs, Peking University, 100191 Beijing, China; 2 Department of Molecular and Cellular Pharmacology, School of Pharmaceutical Sciences, Peking University, 100191 Beijing, China

**Keywords:** Biotechnology, Immunology

**Fig. 1:**
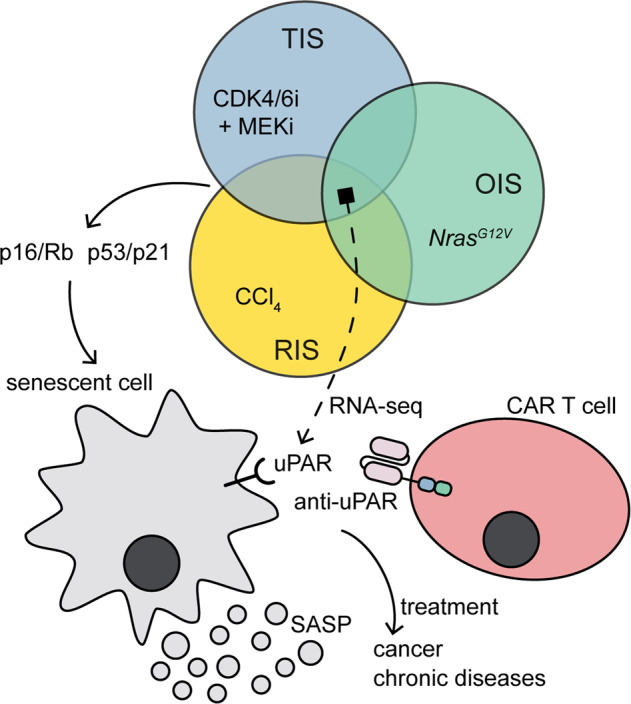
Schematic illustration of uPAR-CAR T-cell therapy. Urokinase-type plasminogen activator receptor (uPAR) was identified as a common upregulated marker in senescent cells by RNA-sequencing (RNA-seq) analysis in three different models: therapy-induced senescence (TIS), oncogene-induced senescence (OIS), and replication-induced senescence (RIS). Chimeric antigen receptor (CAR) T cells targeting uPAR can be utilized for the treatment of cancer and chronic diseases. SASP: senescence-associated secretory phenotype

**A very recent study published in** ***Nature*** **by Amor et al.**^1^ **identifies the urokinase-type plasminogen activator receptor (uPAR) as a broadly induced specific cell-surface marker during cell senescence, which could be used as a target for chimeric antigen receptor (CAR) T cell to specifically ablate senescent cells** **in vitro and** **in vivo. The authors intriguingly show that uPAR-specific CAR T-cell therapy improves the treatment outcome of lung adenocarcinomas and efficiently reduces liver fibrosis in mouse models, thus offering a promising novel therapeutic strategy for senescence-associated diseases (Fig.** **1****)**.

Cell senescence refers to a cell state involving permanent growth arrest, apoptosis resistance, and acquisition of a senescence-associated secretory phenotype (SASP), and can be induced by diverse stress signals, such as DNA damage, reactive oxygen species (ROS), oncogene activation, and proteotoxic stress. Although it plays an important part in embryonic development, wound healing and tumor suppression, senescent cell also has a causative role in chronic diseases and other age-associated pathologies. Senolytic drugs have shown promising results in eliminating senescent cells and alleviating a range of conditions in several animal models. For example, the pan-BCL inhibitor, ABT-263, can selectively eliminate senescent cells from atherosclerotic plaques and brain tissue, thus attenuating the progression of disease phenotype.^2^ Other senolytic agents, such as UBX0101 and UBX1967, are under clinical trials for the treatment of osteoarthritis and eye diseases, respectively.^3^ However, senolytic drugs generally suffer from toxic side effects, due to “off-target” towards other senescence-related processes. Specific cell markers are still under investigation and the next-generation targeted senolytics are now being developed. To this extent, cell therapy such as CAR T-cell therapy might pave a new way for treating senescence-associated diseases by removing senescent cells.

To investigate whether CAR T cells could serve as senolytic agents, Amor et al.^1^ first attempted to identify cell-surface markers broadly and specifically upregulated in senescent cells. By comparing RNA-sequencing (RNA-seq) datasets derived from three senescence models, they found eight gene candidates encoding surface molecules that are commonly upregulated in therapy-induced senescence (TIS), oncogene-induced senescence (OIS), as well as replication-induced senescence (RIS) in hematopoietic stem cells (HSCs). After excluding genes highly expressed in non-senescent cells and in vital tissues, they identified *PLAUR*, which encodes the urokinase-type plasminogen activator receptor (uPAR), as a suitable candidate upregulated both in senescent mouse and human cells. Further experiments were conducted to confirm that uPAR was overexpressed on the surface of senescent cells in senescence-induced mouse KP lung cancer cells in vitro, in mouse models, and in patients-derived tissues with senescence-associated disorders, including liver fibrosis, osteoarthritis, diabetes, and idiopathic pulmonary fibrosis. Collectively, these studies strongly implicated that uPAR is a specific and broadly induced cell-surface marker for senescent cells. The identification of uPAR will be useful not only for providing a specific cell-surface and secreted biomarker for senescence, but also for providing a potential candidate target for generating senolytic CAR T cells.

CAR T-cell therapy has been successfully applied in patients with certain leukemia or lymphoma. In fact, a recent study has generated CAR T cells targeting uPAR for ovarian cancer.^4^ Instead of using single-chain variable fragment (scfv), they used the natural ligand (a part of uPA) of uPAR to demonstrate the efficacy of uPAR-CAR T cells against ovarian cancer cells in vitro. Nonetheless, investigating CAR T-cell therapy in non-oncology study is rare, and certainly not in senescence-associated pathologies. To provide a proof-of-principle study, the authors generated senolytic CAR T cells by introducing anti-mouse uPAR (m.uPAR) scfv linked to human CD28 costimulatory and CD3ζ (h.28z) signaling domains (m.uPAR-h.28z) into human T cells (m.uPAR-h.28z CAR T cells). The resulting CAR T cells showed specific and efficient cytotoxicity towards uPAR-positive senescent cells.

To evaluate the senolytic ability of uPAR-CAR T cells in vivo, oncogene-induced hepatocytes senescence model in immunodeficient NOD scid gamma (NSG) mice were treated with m.uPAR-h.28z CAR T cells, and efficient elimination of senescent cells was observed. In addition, when combined with senescence inducing agents (MEK and CDK4/6 inhibitors) in immunocompetent settings, the fully mouse CAR (m.uPAR-m.28z) T cells showed much improved treatment outcome and such combinatorial strategies could be used against solid tumors. Additionally, the authors explored the applications of senolytic CAR T cells in the treatment of other senescence-related disorders, such as liver fibrosis. CCl_4_-induced and non-alcoholic steatohepatitis (NASH)-induced liver fibrosis were chosen as two models of chronic tissue pathologies. In both models, m.uPAR-m.28z CAR T cells showed efficacy to remarkably eliminate senescent cells, reduce fibrosis, and improve liver function. Altogether, these findings indicated that uPAR-specific senolytic CAR T cells were effective against senescent cells for treatment of cancer, as well as liver fibrosis. It is noteworthy that although treatment with lower effective dose showed no toxicity, mice treated with supratherapeutic dose of m.uPAR-m.28z CAR T cells showed transient hypothermia and weight loss, accompanied by a rise in serum cytokines, including IL-6, GM-CSF, G-CSF, and IFN-γ, which is similar to CAR T-cell-associated cytokine release syndrome (CRS).

In summary, the study by Amor et al. disclosed uPAR as a common upregulated surface marker of senescent cells. Moreover, the authors elegantly demonstrated that uPAR-targeted CAR T cells could specifically and efficiently eliminate senescent cells both in vitro and in vivo, providing an unconventional strategy for the treatment of cancer and senescent-related disorders, such as liver fibrosis. However, the safety profile of uPAR-specific CAR T-cell therapy remains to be clinically developed to minimize its toxicity.^5^ Tightly controls, such as safety switches or combinatorial strategies can later be introduced in order to finely regulate the cytotoxicity of uPAR-targeted CAR T cells. With the development of RNA-sequencing and bioinformatics, more specific and powerful targets may be identified in senescent tissues, and this novel therapy may be further applied to other senescence and chronic-related disorders, like severe atherosclerosis, diabetes, and osteoarthritis.
